# Defining the relationship between *Plasmodium vivax* parasite rate and clinical disease

**DOI:** 10.1186/s12936-015-0706-3

**Published:** 2015-05-07

**Authors:** Katherine E Battle, Ewan Cameron, Carlos A Guerra, Nick Golding, Kirsten A Duda, Rosalind E Howes, Iqbal RF Elyazar, Ric N Price, J Kevin Baird, Robert C Reiner, David L Smith, Peter W Gething, Simon I Hay

**Affiliations:** Spatial Ecology and Epidemiology Group, Tinbergen Building, Department of Zoology, University of Oxford, South Parks Road, Oxford, UK; Sanaria Institute for Global Health and Tropical Medicine, Rockville, MD USA; Wellcome Trust Centre for Human Genetics, University of Oxford, Roosevelt Drive, Oxford, UK; Eijkman-Oxford Clinical Research Unit, Jalan Diponegoro No 69, Jakarta, Indonesia; Global and Tropical Health Division, Menzies School of Health Research, Charles Darwin University, Darwin, NT Australia; Centre for Tropical Medicine and Global Health, Nuffield Department of Medicine, University of Oxford, Oxford, UK; Indiana University School of Public Health, Bloomington, IN USA; Fogarty International Center, National Institutes of Health, Bethesda, MD USA; Institute for Health Metrics and Evaluation, University of Washington, Seattle, WA 98121 USA

**Keywords:** Malaria, *Plasmodium vivax*, Epidemiology, Incidence, Prevalence, Model

## Abstract

**Background:**

Though essential to the development and evaluation of national malaria control programmes, precise enumeration of the clinical illness burden of malaria in endemic countries remains challenging where local surveillance systems are incomplete. Strategies to infer annual incidence rates from parasite prevalence survey compilations have proven effective in the specific case of *Plasmodium falciparum*, but have yet to be developed for *Plasmodium vivax*. Moreover, defining the relationship between *P. vivax* prevalence and clinical incidence may also allow levels of endemicity to be inferred for areas where the information balance is reversed, that is, incident case numbers are more widely gathered than parasite surveys; both applications ultimately facilitating cartographic estimates of *P. vivax* transmission intensity and its ensuring disease burden.

**Methods:**

A search for active case detection surveys was conducted and the recorded incidence values were matched to local, contemporary parasite rate measures and classified to geographic zones of differing relapse phenotypes. A hierarchical Bayesian model was fitted to these data to quantify the relationship between prevalence and incidence while accounting for variation among relapse zones.

**Results:**

The model, fitted with 176 concurrently measured *P. vivax* incidence and prevalence records, was a linear regression of the logarithm of incidence against the logarithm of age-standardized prevalence. Specific relationships for the six relapse zones where data were available were drawn, as well as a pooled overall relationship. The slope of the curves varied among relapse zones; zones with short predicted time to relapse had steeper slopes than those observed to contain long-latency relapse phenotypes.

**Conclusions:**

The fitted relationships, along with appropriate uncertainty metrics, allow for estimates of clinical incidence of known confidence to be made from wherever *P. vivax* prevalence data are available. This is a prerequisite for cartographic-based inferences about the global burden of morbidity due to *P. vivax*, which will be used to inform control efforts.

**Electronic supplementary material:**

The online version of this article (doi:10.1186/s12936-015-0706-3) contains supplementary material, which is available to authorized users.

## Background

Reliable estimates of clinical incidence of malaria have been an enduring challenge for epidemiologists working to measure the impact of the disease, define targets for control, and evaluate progress towards elimination [1-10]. Direct clinical incidence surveys are costly and time-consuming; as a result, many published large-scale estimates of incidence rely on passive reporting of cases to routine health information systems. These data are often incomplete or inaccurate [11,12] and must be adjusted using relationships between variables of unknown certainty [10]. Prevalence, or parasite rate (PR), on the other hand, is a more easily measured and widely available malaria metric [13]. A species-specific modelled relationship between *Plasmodium vivax* PR (*Pv*PR) and the rate of clinical illness, similar to that developed for *Plasmodium falciparum* [5,14], would be an important step towards the generation of a continuous global map of *P. vivax* burden from which national and sub-national aggregate estimates of annual incidence can be compiled with known uncertainties.

Enumeration of the global disease burden attributable to *P. vivax* malaria has been identified as a key knowledge gap [15-17]. Large discrepancies exist in the currently available burden estimates [18], which have been calculated using a variety of methods. Figures based on cases reported to health systems estimate *P. vivax* incidence to be 15.8 million cases per year [2,18]. However, estimates derived from the ‘cartographic’ approach using mapped endemicity classes and populations at risk suppose that these values would be far greater: 132-391 million cases annually [4,17]. The cartographic method bypasses some of the challenges inherent in the surveillance-based approach, in which the numbers of cases reported are adjusted to account for incompleteness in reporting, usage of health facilities, and diagnostic confirmation, and it is difficult to quantify the precision of these adjustments. The cartographic approach, on the other hand, estimates cases through a geostatistical model of endemicity constrained by the input data, with strength borrowed implicitly from observations at neighbouring sites, such that the resulting case estimates carry a formal uncertainty metric testable via cross-validation. Both techniques have their limitations and reconciling them is a long-term goal; the first step towards which is a fuller implementation of the cartographic approach for *P. vivax*.

**Figure 1:**
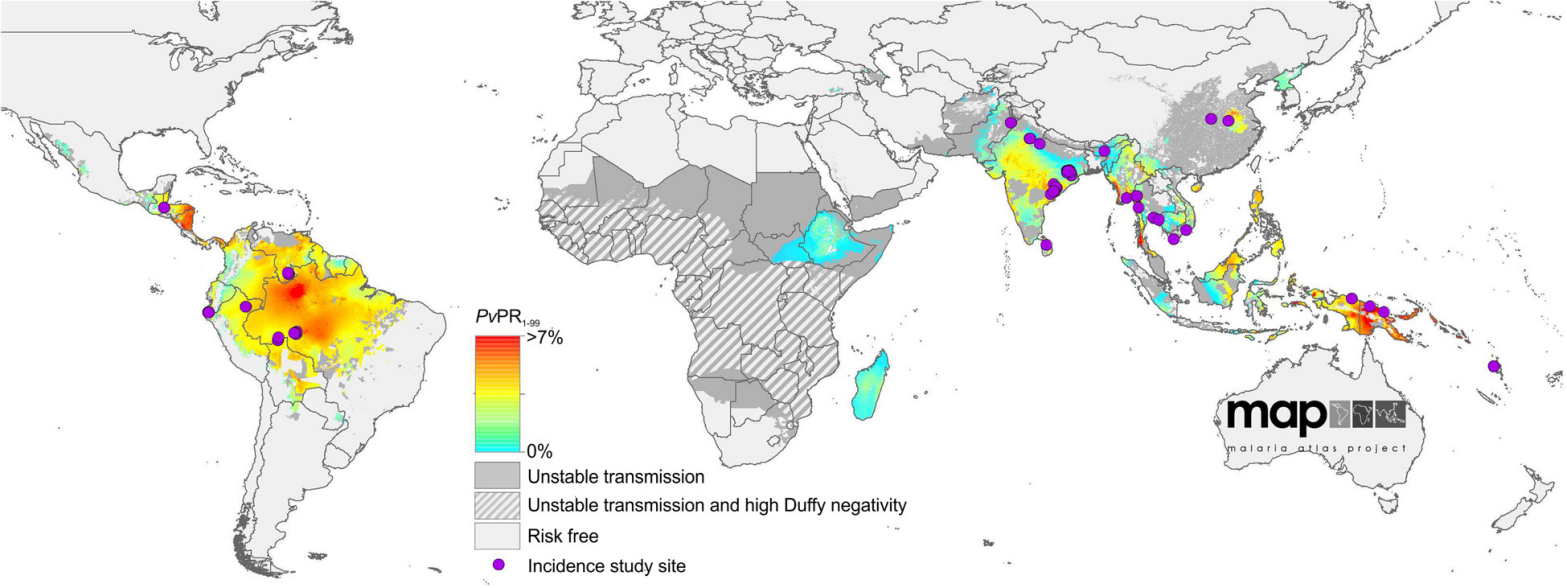
The spatial distribution of *Plasmodium vivax* endemicity in 2010 overlaid by ACD study sites. The spatial distribution of *P. vivax* [19] is shown using the MBG point estimates of the annual mean *Pv*PR (1 to 99 year-olds) within the spatial limits of stable transmission, displayed on a continuum of blue (low prevalence) to red (high prevalence). Areas within the stable limits that were predicted with high certainty (>0.9) to have a *Pv*PR less than 1% were classed as unstable. Regions where Duffy negativity gene frequency is predicted to exceed 90% [42] are shown in hatching for additional context. The location of study sites of the incidence records used in the final analysis are shown as purple points.

A global map of *P. vivax* prevalence from which cartographic incidence estimates may be generated has been published for 2010 [19] and efforts to update this map are underway. The 2010 map, shown in Figure 1, displays the stable and unstable limits of transmission as defined according to annual parasite incidence (API) data, as well as the predicted *Pv*PR (as a population average over the one to 99 year-old age range) at a 5 × 5 km pixel scale within the stable limits of *P. vivax* transmission (API ≥0.1 per 1,000 per annum) [20]. As this map illustrates, large swaths of densely populated areas are exposed to stable transmission, though it remains unclear how many clinical infections arise from the 2.5 billion people who live within the limits of *P. vivax* transmission [21] because the relationship between *Pv*PR and incident morbidity has not yet been reliably established for *P. vivax*.

**Figure 2:**
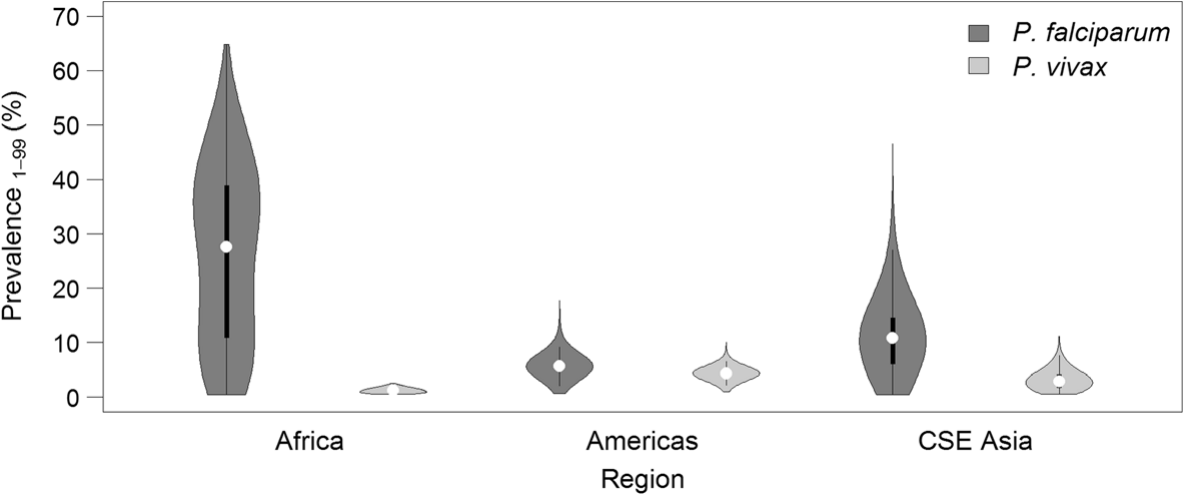
Comparison of *Plasmodium falciparum* and *Plasmodium vivax* prevalence. Prevalence values, obtained from the mapped *P. falciparum* and *P. vivax* endemicity surfaces [19,24]. Data for *P. falciparum* has been standardized to the 1 to 99 years age range to reflect *P. vivax* data [36]. The shaded areas correspond to each species and show a smoothed approximation of the frequency distribution (a kernel density plot) of parasite prevalence within each geographic region. The black central bar represents the interquartile range and the white circles indicate the median values.

It is necessary to model *P. vivax* separately from *P. falciparum* because of the biological and epidemiological differences that affect their observed prevalence of infection and patterns of clinical incidence [22]. Vivax malaria circulates in the blood at much lower parasite densities than *P. falciparum*, making it less likely to be detected by diagnostic techniques commonly used to measure prevalent infections: light microscopy and rapid diagnostic tests (RDTs) [22]. Nevertheless, low blood-parasite densities are still able to elicit symptomatic disease [23]. Cartographic estimates of *Pv*PR are an approximate order of magnitude lower than those for *P. falciparum* [19,24]. Although prevalence values exceeding those shown on the scale in Figure 1 are observed, particularly among children (see associated dataset [25]), the community prevalence of *P. vivax* is consistently low relative to *P. falciparum*, as illustrated in Figure 2. A possible explanation for this effect is natural immunity, which is acquired more rapidly against *P. vivax* than *P. falciparum*, such that infection prevalence peaks in young children, with *Pv*PR in adults significantly lower [22]. Prevalence of *P. vivax* starts to decline after the second year of age, whereas *P. falciparum* prevalence continues to rise until later in life in all but the most intense transmission settings [26,27].

The most significant biological difference between *P. vivax* and *P. falciparum* is the ability of *P. vivax* to form liver stages capable of causing relapsing infections weeks to months after the initial inoculation [28]. Hence, in contrast to *P. falciparum*, which has only sporozoite-induced infections, blood-stage parasitaemia in *P. vivax* can arise from either mosquito-borne sporozoites or liver-borne hypnozoites. This has significant consequences for measuring the force of infection of blood-stage *P. vivax*. However, for the purpose of estimating the burden of clinical disease, the origin of the infection is not of utmost significance. A primary mosquito-borne infection and a relapsing hypnozoite-borne infection are both capable of causing symptomatic illness as well as onward transmission, and incidence from both are correlated with parasite prevalence. Hence, this study did not attempt to differentiate the incidence of relapse from the incidence of new infections, but rather examined the relationship between prevalent parasitaemia and incidence of clinical disease by geographic regions stratified by differing relapse patterns [28].

Issues inherent in estimating the burden of *P. vivax* malaria are addressed here by defining the relationship between published symptomatic *P. vivax* incidence rates derived from active case detection (ACD), matched with age-standardized measures of infection prevalence.

## Methods

### Data assembly

The aim of the data assembly was to build a comprehensive database of reports of clinical (symptomatic) incidence of *P. vivax* measured by ACD since 1 January, 1985, to be consistent with the *Pv*PR data used to develop global endemicity maps. A formal literature search was conducted in PubMed [29] on 27 November, 2013 using the search terms: ((malaria[MeSH Terms]) AND (“Incidence” [Mesh] OR “Epidemiology” [Mesh] OR “epidemiology” [Subheading])) AND (“1985/01/01”[Date - Publication] : “3000”[Date - Publication]). This returned 11,272 references.

Abstracts of all references returned were reviewed to determine if clinical incidence data could potentially be included in the paper. Reviews, case studies, and reports on imported malaria, animal studies, vector-only studies, and technical analyses (such as genetic mapping or transmission models) were excluded at this stage. Studies that did not explicitly report *P. vivax* incidence data collection in the abstract were not excluded in case it was reported in the main body.

The full texts of the 898 selected references, plus 78 publications flagged from previous studies [14,30], were then checked for the following criteria: (i) they contained longitudinal survey data involving ACD of symptomatic cases (typically defined by presence or recent history of fever); (ii) they were conducted in the general community (i.e., not patient sub-groups); (iii) malaria was diagnosed using microscopy or RDTs; and, (iv) results were presented in such a way that the number of cases and person-time observed could be determined. Due to diagnostic limitations, cases could not be distinguished as hypnozoite-borne and sporozoite-borne infections. There were no restrictions placed on age of the study population. For the initial data extraction, no limit was placed on the length or regularity of ACD, as long as the case detection methods were specifically reported. Studies that used passive case detection (PCD) only or were cross-sectional surveys were excluded.

Studies were geopositioned to a region (Africa, Americas or Central and Southeast (CSE) Asia), country and place name, and mapped to a specific latitude and longitude using location information from the source and gazetteers such as Encarta [31] and Google Maps [32], as described previously [33]. The studies were also classified to a geographic zone of relapse phenotype as defined by Battle *et al.* [28]. Patterns in the timing of the first relapse event are thought to vary geographically among the zones illustrated in Additional file 1: Figure S1. The size and age range of a study cohort was extracted, and a single study reporting on multiple age ranges was disaggregated into separate records. Likewise, if a study contained different treatment or intervention arms, these were entered as separate records, and any control methods in place separate from the study were noted accordingly. Details regarding the time and length of the survey were recorded, along with type (ACD only or ACD + PCD) and frequency of detection. The number of cases and the person-years observed were recorded to determine incidence, as well as the diagnostic method, case definition, and any parasite density threshold applied to that definition.

If the number of person-years observed was not reported, it was estimated by multiplying the population of the study cohort by the length of the study. As this method of estimation may over-estimate person-time due to study members being lost to follow-up (and therefore under-estimate incidence), it was recorded whether person-time was explicitly reported in the study or if it had to be estimated.

### Matching incidence to prevalence

Where possible, *Pv*PR data were extracted from the same publication as the incidence data to provide a temporally matched measure of prevalence in the same community. If *Pv*PR data were not reported, the Malaria Atlas Project (MAP) database [33,34] was searched for a prevalence study conducted in the same community in the same time period as the incidence study. For the records without a matched *Pv*PR value, a predicted prevalence was extracted from the *P. vivax* MAP endemicity surface using ArcGIS [35]. The methodology used to generate this surface is described in detail elsewhere [19,20], but briefly: the predicted *Pv*PR values represent an annualized mean prevalence in all ages (1-99 years) drawn from a species-specific model-based geostatistical (MBG) framework using 9,970 *Pv*PR surveys collected from 1985 to 2010 plus a suite of environmental covariates to estimate the prevalence in every 5 × 5 km square within the limits of stable transmission.

**Figure 3:**
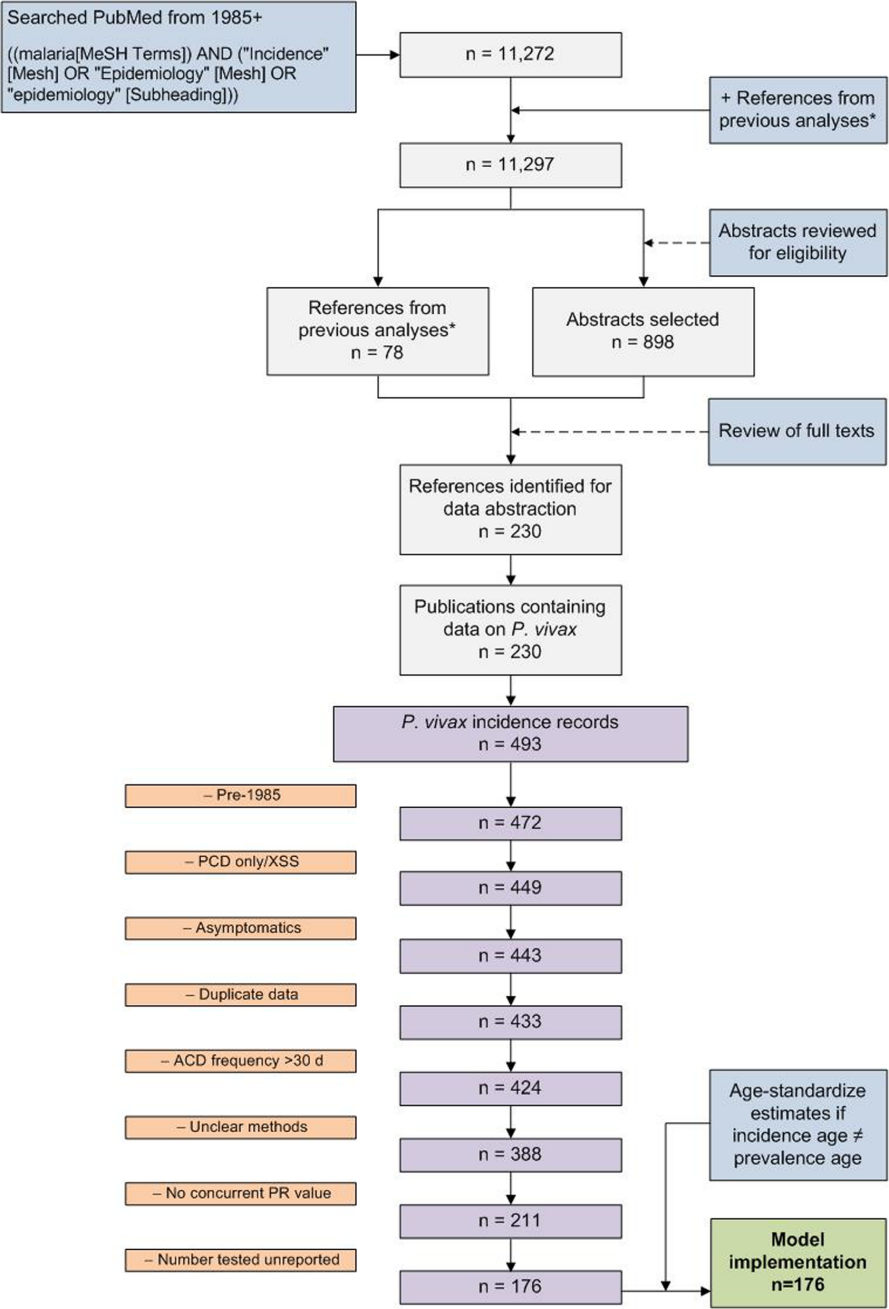
Schematic overview of the literature search procedure, results, and data exclusions to obtain clinical incidence records of use for model implementation. References from previous analyses* include those used by Patil *et al*. [14] and Griffin *et al*. [30].

To facilitate modelling of the prevalence – incidence relationship, each inferred prevalence was standardized to a common age range of 0 to 85 years using the age-standardization model developed by Smith *et al.* initially for *P. falciparum* [36] and later updated for *P. vivax* [19]. The age-standardization was implemented using a freely available software package developed by the authors for the R statistical programming environment [37,38]. The full dataset used in this study and further details regarding its assembly are available in a dedicated publication [25]. A schematic of the data assembly stages is shown in Figure 3.

### Model development

A Bayesian hierarchical model was developed to describe the relationship between the population prevalence and clinical incidence of vivax malaria. The model included a composite likelihood function to account for various aspects of the data: (i) the inherent randomness of the standard sampling distributions for both the parasite positive count (binomial) and the clinical case count (Poisson) at each site; (ii) a potential over-dispersion (extra-Poissonian variance) in the incidence observations attributable to site and study-specific random effects; (iii) a dependence of observed clinical incidence on the frequency of ACD [39]; and (iv) the impact of variation in the range of ages targeted by each study design given the importance of exposure-based, and hence age-dependent, immunity to clinical illness. While the asynchronous sampling of incidence and prevalence in different transmission seasons evinced by some surveys was not modelled explicitly, its contribution to the observational variance was effectively allowed for by these study- and site-specific random effects terms.

**Figure 4:**
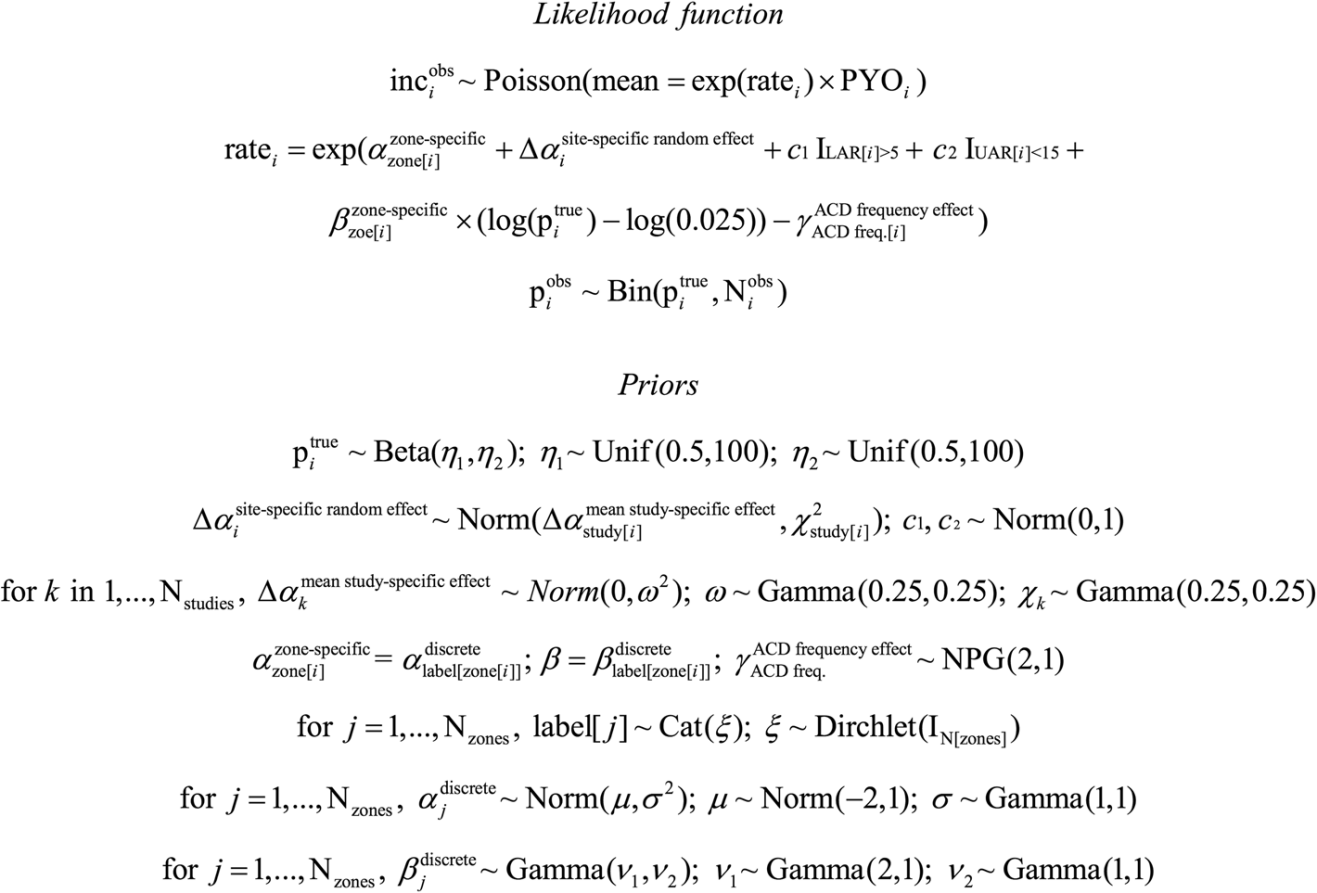
The mathematical form of the model summarized in standard hierarchical Bayesian notation.

To account for (iii), in the absence of a single widely accepted parametric model of the effect of ACD occurrence, a non-parametric approach was used to infer this relationship. A modular statistical distribution was defined over the space of monotonically decreasing functions evaluated at the seven unique regularities of detection used in the ACD studies in the database: daily, every other day, every third day, five times per week, weekly, fortnightly, and monthly. The generative model for this distribution (denoted in Figure 4 as non-parametric gamma) was defined with respect to the joint order statistic of seven random variables; each gamma-distributed with a shape parameter of two and a rate parameter of one.

To account for (iv) a pair of scale coefficients were introduced to the likelihood function and fit simultaneously with all other random effects: the first acting to scale down the expected incidence for studies excluding children below 5 years of age, the second acting to scale up that for studies excluding older children and adults above 15 years of age. The age standardization of contemporary prevalence estimates described above was propagated to the input data as an adjustment of the numerator (observed parasite positives) in the binomial likelihood.

The fitted model for the prevalence – incidence relationship adopted here was a linear regression of the logarithm of the incidence rate against the logarithm of prevalence with zone-specific clustering effects. Note: as standard terminology in the statistical literature, the term ‘linear’ here denotes linearity in the coefficients, not necessarily the explanatory variables on which they act. Up to six unique intercepts and slopes were allowed to represent the six geographically bounded relapse phenotype zones represented in the dataset. Since variation in the epidemiology of *P. vivax* is not strictly defined by the geographic divisions proposed, the model was allowed to fit multiple zones with a common prevalence – incidence relationship by labelling these six possible slope-intercept pairs in order of increasing slope and treating label assignment for each zone as a categorical variable with proportions assigned a Dirichlet prior. Hence, multiple zones may share the same intercept and slope, and thereby share power for their inference, where this ‘clustering’ scenario is consistent with the observed data.

The mathematical form of this model is summarized in standard hierarchical Bayesian notation in Figure 4. Posterior simulation for this model was achieved via rejection Gibbs sampling with the JAGS (Just Another Gibbs Sampler) software package [40], with data entry and graphical summary achieved via the R statistical computing environment [37].

## Results

### Data assembly

**Figure 5:**
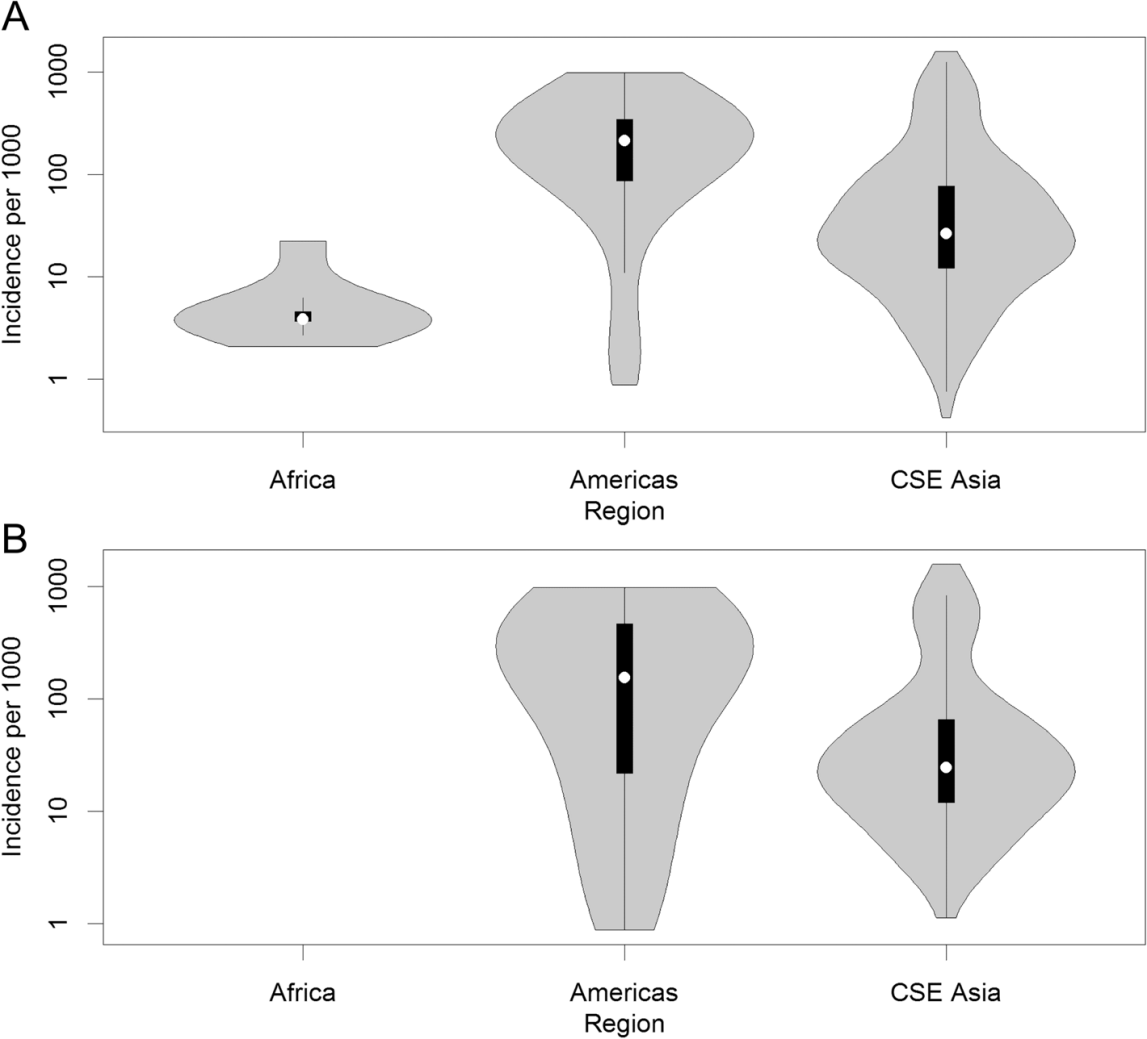
Violin plot of incidence (per 1,000 person-years observed). **A)** all data (n = 388) by region and **B)** data used in the analysis (n = 176) by region are shown with incidence on the logarithmic scale. The grey areas correspond to a smoothed approximation of the frequency distribution (a kernel density plot) of the incidence observed in each geographic region. The black central bar represents the interquartile range and the white circles indicate the median values.

*Plasmodium vivax* clinical incidence data were identified in 99 publications. Following checks that the studies met the inclusion criteria described above, these data were abstracted into 388 reports of incidence. The majority of the data came from CSE Asia (80%, 311/388), as shown in Table 1, with ten records from Africa and 67 from the Americas. Data originated from 18 countries in total: ten from CSE Asia, five from the Americas and three from Africa. The incidence measures observed ranged from zero to 1.6 per person year observed. The highest incidence values observed were in CSE Asia in Papua New Guinea (PNG). Summary statistics of the incidence observed by MAP region are shown in Table 2, and the violin plots in Figure 5.

**Table 1:** **Data records by MAP region**

**Region**	**All ** ***P. vivax *** **data**	**Data used in model**
Africa+	10	0
America	67	43
CSE Asia	311	133
**Total**	**388**	**176**

**Table 2:** **Incidence summary statistics**

**All data - incidence per 1,000 person-years observed**							
**Zone**	**Zone name**	**N**	**Minimum**	**Mean**	**Median**	**Maximum**	**IQR**
**2**	**Central America**	3	72.07	103.93	80.00	159.71	43.82 (76.04, 119.86)
**3**	**South America**	64	0.00	227.47	161.52	977.31	281.06 (40.33, 321.39)
**7**	**Sub-Saharan Africa**	10	0.00	4.99	3.75	22.19	1.45 (2.48, 3.93)
**8**	**Monsoon Asia**	265	0.00	42.49	20.24	412.87	49.29 (8.05, 57.34)
**10**	**Southeast Asia**	24	0.00	291.56	290.87	710.50	497.62 (28.17, 525.79)
**11**	**N. Europe and Asia**	4	20.32	33.30	35.05	42.78	6.61 (30.87, 37.48)
**12**	**Melanesia**	18	56.81	709.63	758.19	1586.07	368.75 (531.25, 900.00)
**All**	**Total**	388	0	118.8	29.82	1586.07	99.38 (9.82, 109.20)
**Data with concurrent ** ***Pv*** **PR values used in analysis - incidence per 1,000 person-years observed**							
	**Region**		**Minimum**	**Mean**	**Median**	**Maximum**	**IQR**
**2**	**Central America**	3	72.07	103.93	80.00	159.71	43.82 (76.04, 119.86)
**3**	**South America**	40	0.00	236.51	138.47	977.31	329.76 (22.16, 351.92)
**8**	**Monsoon Asia**	100	0.00	26.51	18.58	194.59	27.56 (7.36, 34.92)
**10**	**Southeast Asia**	18	4.48	250.68	89.77	692.31	472.71 (25.92, 498.63)
**11**	**N. Europe and Asia**	4	20.32	33.30	35.05	42.78	6.61 (30.87, 37.48)
**12**	**Melanesia**	11	56.81	674.07	658.76	1586.07	492.43(400.00, 892.43)
**All**	**Total**	176	0	139.10	29.10	1586.07	87.18 (11.74, 98.92)

### Matching incidence to prevalence

Slightly less than half of the records (46%, 180/388) had a prevalence value available from the same reference. An additional 31 prevalence values were added to records using entries in the MAP database that were collected in the same site during the same year. This provided a space-time matched PR for approximately half (54%, n = 211) of the incidence records. A *Pv*PR value for each of the remaining 177 incidence records was obtained from the MAP *P. vivax* endemicity surface [19]. The MAP-based *Pv*PR values represent all-age estimates, and 123 (69%) of the incidence records without concurrent *Pv*PR were also measured in all ages. Of the incidence records with a concurrent *Pv*PR estimate, 154 (73%) of *Pv*PR surveys were conducted in the same age group as the ACD cohort. The 110 *Pv*PR values that were not age-matched to the incidence data were age-standardized to the same age-range as the incidence data [36,38]. The *Pv*PR estimates for all records ranged from zero to just over 30%. The highest estimates were again observed in PNG. *Pv*PR data summary statistics are shown in Table 3.

**Table 3:** **Parasite rate (%) summary statistics**

**All data - using concurrent ** ***Pv*** **PR or MAP-based ** ***Pv*** **PR from the ** ***P. vivax *** **endemicity map**							
**Zone**	**Zone name**	**N**	**Minimum**	**Mean**	**Median**	**Maximum**	**IQR**
**2**	**Central America**	3	1.1.6	1.28	1.27	1.40	0.12 (1.22, 1.34)
**3**	**South America**	64	0.00	2.51	1.80	7.65	3.41 (0.89, 3.36)
**7**	**Sub-Saharan Africa**	10	0.47	0.66	0.45	1.67	0.00 (2.48, 3.93)
**8**	**Monsoon Asia**	265	0.00	2.98	2.68	30.88	1.48 (1.26, 3.90)
**10**	**Southeast Asia**	24	0.00	3.39	3.43	6.98	3.47 (1.33, 4.42)
**11**	**N. Europe and Asia**	4	0.45	1.50	1.73	2.09	0.53 (2.11 2.94)
**12**	**Melanesia**	18	2.92	11.81	10.92	28.41	6.93 (9.82, 16.61)
**All**	**Total**	388	0.00	3.25	2.61	30.88	2.35 (1.27, 3.62)
**Data with age-matched concurrent ** ***Pv*** **PR used in the analysis**							
	**Region**		**Minimum**	**Mean**	**Median**	**Maximum**	**IQR**
**2**	**Central America**	3	1.16	1.27	1.27	1.40	0.12 (1.22, 1.34)
**3**	**South America**	40	0.00	1.41	0.89	7.52	1.94 (0.00, 1.94)
**8**	**Monsoon Asia**	100	0.00	2.92	2.14	12.59	2.98 (0.90, 3.88)
**10**	**Southeast Asia**	18	0.79	2.98	2.14	6.98	3.09 (1.33, 4.42)
**11**	**N. Europe and Asia**	4	0.71	2.35	2.71	3.27	0.83 (2.11, 2.94)
**12**	**Melanesia**	11	8.25	14.52	14.77	28.41	6.04 (8.25, 15.95)
**All**	**Total**	176	0.00	3.27	1.87	28.41	3.05 (0.84, 3.89)

**Figure 6:**
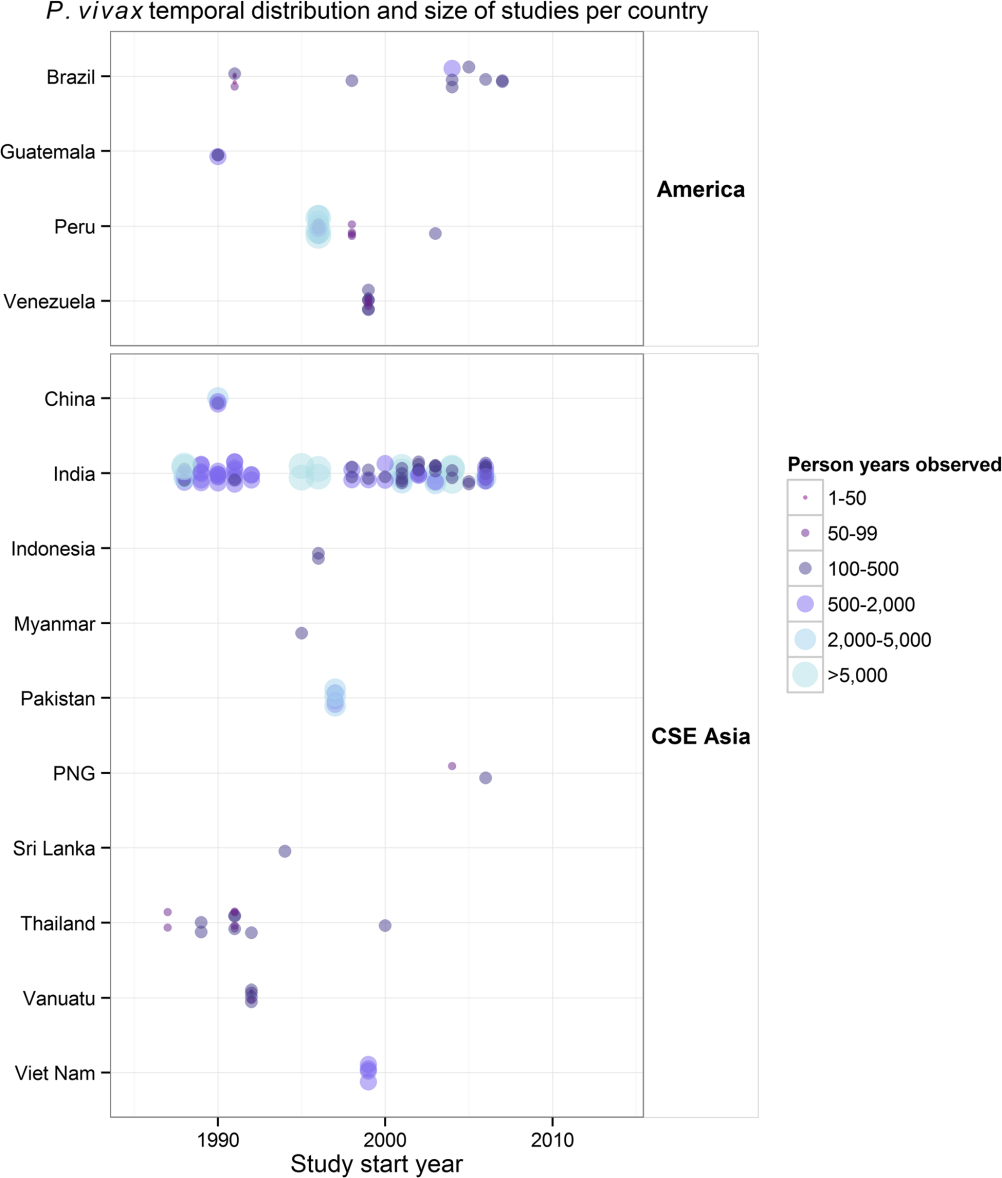
Temporal distribution of records used in the analysis. The size of the point reflects the number of person-years observed included in the 176 records that had an age-matched concurrent *Pv*PR measure with the incidence record.

The prevalence values extracted from the *P. vivax* endemicity map had a similar range to the *Pv*PR estimates measured alongside incidence (from close to zero to ~25%), but less variation (Additional file 2: Figure S2). This was because multiple incidence records that came from the same or nearby locations were matched to a *Pv*PR from the same or similar pixels in the predicted *Pv*PR map. Statistically, incidence records with only *Pv*PR values derived from the map were excluded because their uncertainties (in part due to the mismatch between the scale of MAP pixels and the scale of *Pv*PR variation within a pixel) were so large that the inclusion of these points did not add to the model fits. That is, only concurrently measured *Pv*PR values – reported from the same reference or another paper from the MAP database – were used. This also facilitated development of the statistical model as the selected studies all presented counts of the number examined and positive, and thus the same type of uncertainty was manifest for both the incidence and *Pv*PR estimates used in the analysis, whereas this would not be true for excluded *Pv*PR surveys that did not report the numerators or denominators (n = 35). Following all exclusions (Figure 3), 176 records from 75 sources remained to be used in the analysis. The temporal distribution and study size, based on person-time observed, of these records are shown in Figure 6.

The approximation of person-time in the majority of the selected records (76%, 133/176) was determined not to be an exclusion criterion. As illustrated in Additional file 3: Figure S3, there was a comparable level of noise in both those records with exact and approximate reported person-time. The data are plotted on both standard and logarithmic scales to also demonstrate that using the logarithm of incidence against the logarithm of prevalence better represents the distribution of the data. Note, however, that only 151 points appear in those panels using logarithmic scales because 25 records had a value of zero (four incidence and 21 prevalence) and could not be readily plotted on these axes.

A specific parasite density threshold in the case definition used in ACD studies was also set aside as an exclusion criterion. The majority of studies specified a case of *P. vivax* as a symptomatic episode at any detectable level of parasitaemia (≥1 asexual stage parasite per μl of blood), but nine of the 176 records included in the analysis specified a parasite density cut-off. Seven records were from studies applying a parasite density threshold of 500 parsites/μl to limit their Type II error rate (false attribution of vivax causality to a background fever) and an additional two applied a cut-off 1,000 parasites/μl. These studies were expected to return lower incidence estimates, but in fact were not observed to be outliers here, as seen in Additional file 4: Figure S4. That the application of the cut-off did not result in lower estimates suggests that vivax-targeting ACD studies are less sensitive to case definition than is the experience for falciparum [41].

### Model development

**Figure 7:**
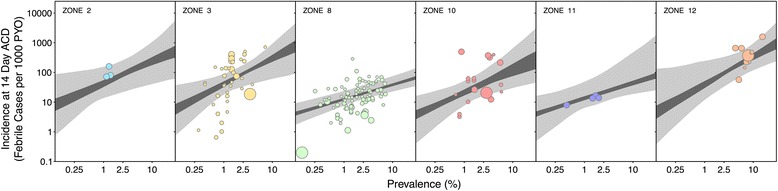
The zone-specific prevalence-incidence relationships shown as point-wise 68% and 95% credible intervals. Zone 2 is Central America, zone 3 is South America, zone 8 is Monsoon Asia (India), zone 10 is Southeast Asia, zone 11 is northern Asia and Europe and Zone 12 is Melanesia. The 95% CrIs are shown in light grey and the 68% CrIs are shown in dark grey. The size of the point corresponds to the time period between each ACD visit (see Figure 8) and the colours of the zones correspond to those shown in Figure 9.

**Figure 8:**
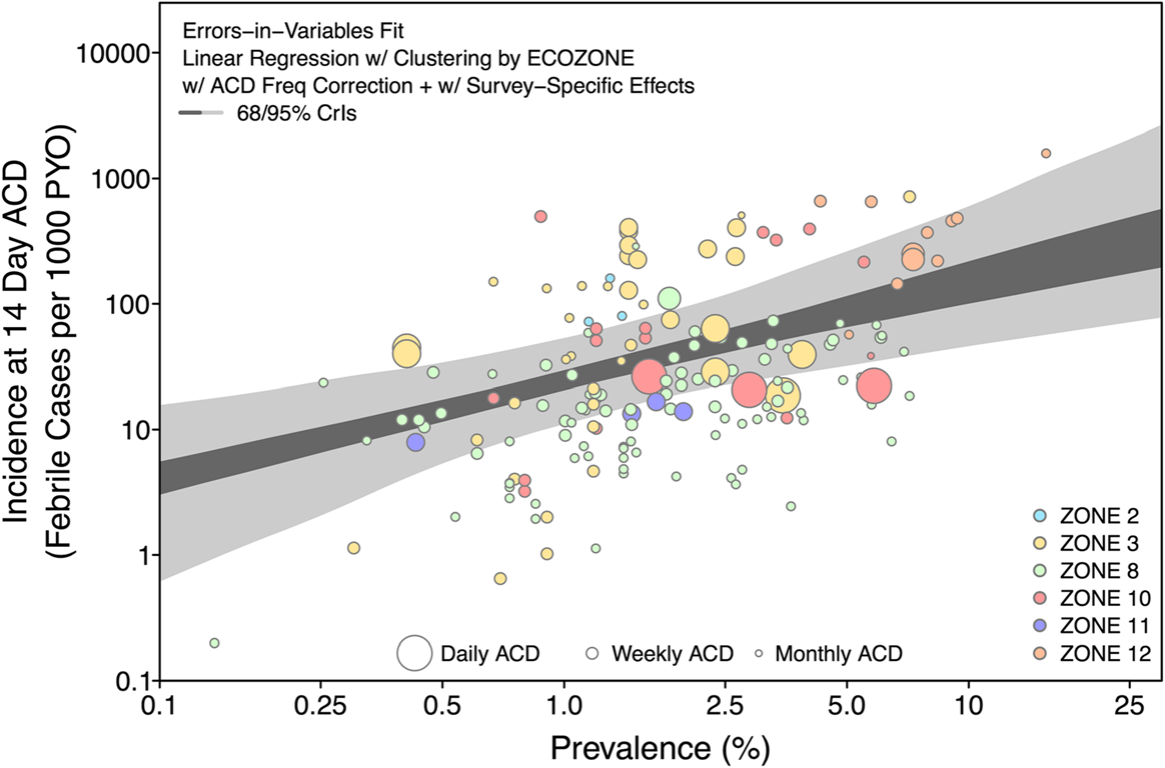
Pooled prevalence-incidence relationship for the entire dataset. To produce a pooled fit, the posterior of each zone was weighted by the number of observations from that zone. An errors-in-variables fit was used to allow for uncertainty in the independent variable as well as the dependent variable (ordinary linear regression would assume no uncertainty in the former). Point-wise 95% CrIs are shown in light grey and 68% CrIs are shown in dark grey. The colours of the zones match those shown in Figure 9.

The posterior for the non-parametric fitted function modelling the impact of ACD regularity on the rate of detected clinical incidence cases is illustrated in Additional file 5: Figure S5. In the subsequent Figures 7 and 8 the (point-wise) mean of this function was used to correct all observed incidence counts to a benchmark of fortnightly ACD. It was estimated that daily ACD studies report on average 12.2 (2.7,42) times (median and 95% credible interval, CrI) the number of fevers identified in studies with fortnightly ACD, whereas the scaling from fortnightly to monthly ACD is less marked at 0.81 (0.34,0.99). Some degree of variation in the dependence of observed incidence on ACD regularity among the geographic zones was expected, such that frequency of ACD would have a greater effect in areas with high risk of recurrence. The model of a shared effect was deemed sufficient, however, because a re-fit of the model allowing each zone to be assigned to one of two separate relationships failed to identify any significant difference in the resulting prevalence-incidence relationship.

The broad posterior credible intervals for the pair of scaling coefficients used here to account for age-dependence of the clinical incidence rate (namely, -0.28 [-0.83,0.27] for *c*_1_ and 0.08 [-0.40,0.66] for *c*_2_) suggest that these terms do not play a crucial role in these fits, a conclusion supported by visual inspection of the zone-specific prevalence – incidence relationships inferred upon exclusion of these terms from the model. However, the consequent inference that exposure-based immunity is unimportant for vivax malaria should be taken with caution: rather the present dataset is underpowered to investigate this effect since over 75% of the studies included here effectively report an all-ages incidence estimate.

**Figure 9:**
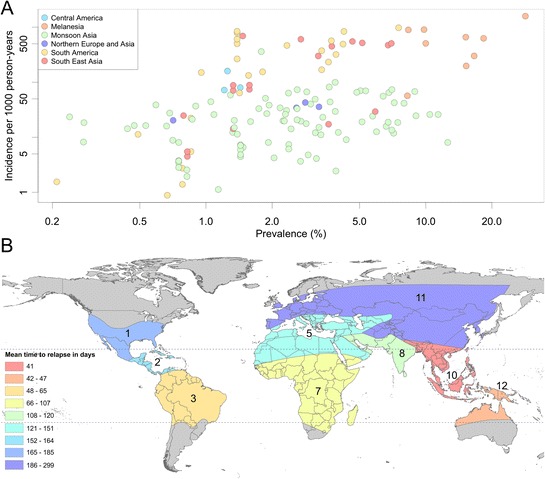
Scatter plot of data used in analysis coloured by relapse zones. Panel **A** plots the data used in the analysis by the relapse zones on log scales. The points are coloured by the mean time to relapse predicted in each zone shown in panel **B**.

The geographic origin of the studies was, however, of importance in the prevalence – incidence model. Figure 9 illustrates the distribution of the matched incidence and prevalence records shaded by the mean time to first relapse in each geographic zone. A significant degree of clustering between zones was identified through the fitted model. In particular, zones 8 and 11 (Monsoon Asia, and northern Asia and Europe) were found to share a common relationship in 50% of the posterior samples. These zones are characterized by long-latency relapse phenotypes (zone 11) or a combination of short and long latency (zone 8). At least three of the four remaining zones (2 - Central America, 3 - South America, 10 - Southeast Asia, and 12 - Melanesia) share a common relationship at a comparable rate. The zone-specific *Pv*PR and clinical incidence relationships thus recovered are illustrated as point-wise 68 and 95% CrIs in Figure 7 and their parameter estimates are summarized in Table 4. In the Table, α is the natural logarithm of incidence per person-year observed at a prevalence of 2.5%; in the model this is the intercept of the (logarithm of) prevalence – (logarithm of) incidence curve, such that the exponent of α is the intercept in cases per person year. Accordingly, β in Table 4 is the slope of the curve, such that if the prevalence were to increase from 2.5 to 7%, the incidence would increase by a factor of exp(β). By weighting the posterior for each zone by the proportion of observations from that zone in the dataset, a pooled relationship was produced for the entire dataset, as illustrated in Figure 8. For reference, the corresponding aggregate parameter estimates of the pooled relationship are α = -3.0 (-3.5,-2.4) and β = 0.71 (0.41,1.10). In other words, based on the pooled relationship a prevalence of 2.5% would correspond to an incidence of 49.8 cases per 1,000 person years (see Table 4).

**Table 4:** **Parameter estimates by zone**

**Zone**	**Name**	**α Median [95% CrI]**	exp(α)*1000 (cases per 1000 person years at 2.5% ***Pv*** **PR)**	**β Median [95% CrI]**
2	Central America	-2.4 [-3.8,-1.4]	90.7	0.68 [0.13,1.53]
3	South America	-2.4 [-3.4,-1.7]	90.7	0.85 [0.29,1.51]
8	Monsoon Asia	-3.9 [-4.4,-3.3]	20.2	0.49 [0.30,0.70]
10	Southeast Asia	-3.1 [-4.1,-2.1]	45.0	0.71 [0.24,1.49]
11	N. Europe and Asia	-3.8 [-4.6,-2.3]	22.4	0.51 [0.18,1.25]
12	Melanesia	-2.4 [-3.4,-1.1]	90.7	0.91 [0.17,1.55]
All	Pooled relationship	-3.0 [-3.5, -2.4]	49.8	0.71 [0.41,1.10]

The results benefit from the model structure by producing associated measures of uncertainty. As shown in Figures 7 and 8, the point-wise CrIs are narrowest around the axis of the regression model at 2.5% prevalence. Zone-specific relationships informed by few data points (zones 2, 11 and 12) have wider CrIs. Based on these wide uncertainty bands, the predicted incidence can change by a factor of 100. While this appears to be a large range, it is representative of the data; Figure 8 illustrates the wide range of incidence measures that were observed in communities with nearly the same *Pv*PR.

## Discussion

This study provides a fundamental component for calculation of the *P. vivax* clinical burden. The result of the work presented here is a model of the relationship between incidence of symptomatic vivax malaria and prevalence of detectable blood-stage *P. vivax* infection. This relationship will allow for the burden of *P. vivax* to be estimated using an updated map of *P. vivax* endemicity. Estimates of burden from maps of prevalence allow for measures of incidence to be made with associated measures of uncertainty.

Two key aspects of the analysis presented are the spatial and temporal components of the data. All incidence data were matched to *Pv*PR data that were measured in the same community at the same time. However, there were subtle differences in how *Pv*PR was measured among the various studies. In many studies, *Pv*PR was measured at the start of the ACD observation period as a baseline measure of endemicity. In other studies, there was more than one cross-sectional survey done during the incidence follow-up period. In those records, the *Pv*PR value is a pooled estimate, which was deemed acceptable because none of the studies administered a radical cure following the initial prevalence survey. This would have contributed to the noise observed in the data, but it is accounted for in the resulting models within the study-specific random effects as well as the uncertainty reflected in the CrIs.

Modelling the relationship between prevalence and incidence specifically for *P. vivax* presented new challenges not encountered in similar work for *P. falciparum* (Ewan Cameron, personal communication, 2015) [14]. There were far less incidence data available for *P. vivax* relative to *P. falciparum* [25]. The majority of the published *P. vivax* incidence data was from CSE Asia. This signals the need for improved active surveillance coverage in the Americas and implementation of RDTs that test for non-falciparum species in areas previously thought to be non-endemic for *P. vivax*, such as East Africa.

There were not age-stratified data available that would have allowed for age-specific burden modelling as done recently for *P. falciparum* (Ewan Cameron, personal communication, 2015). Age-dependent immunity causes high incidence of infection in very young children in high transmission settings with lower incidence in older children and adults [22]. Over 75% of the studies used in the analysis were conducted in whole populations, but the differing age groups in the remainder of the dataset was dealt with through a statistical correction designed to scale down the expected incidence in populations that did not include young children (under five years) and scale up the incidence in populations that did not include children and adults over 15 years of age. Further work involving this model will be improved as *P. vivax* transmission models are developed and the dependence of infection on age in different transmission settings can be explicitly derived.

Aside from issues of data availability, biological features of *P. vivax*, including its ability to cause relapsing infections following an initial mosquito-borne infection, were by necessity treated somewhat pragmatically in this modelling exercise. That is, relapse was not explicitly incorporated into the model since clinical cases due to relapse are captured by both the incidence and prevalence data. Rather, zone-specific relationships were developed to account for varying geographic patterns of relapse [28]. The slope of the prevalence-incidence relationship curve was steeper in regions where relapse is observed to occur rapidly following the primary infection. Zones with long latency relapse phenotypes, and therefore reduced annual relapse incidence (Figure 9 and Table 4), such as Monsoon Asia and northern Europe and Asia, show shallower slopes. These regions, as shown in Figure 7, reach an incidence of one case per 100 people per year at around 1% prevalence, whereas the other regions shown reach a similar incidence level at even lower prevalence values.

## Conclusion

The modelling outputs presented here inform the understanding of the nature of prevalence and incidence relationships, but more importantly, the zone-specific relationships will facilitate global predictions of clinical burden to be made that account for regional differences in *P. vivax* epidemiology. Because of its ability to relapse, *P. vivax* will be the final hurdle as regions move towards elimination in much of the malaria-endemic world. Burden estimates of known accuracy will enable assessments to be made of the impact of *P. vivax* malaria on health systems and economies within and among endemic regions, which will be essential to strategic planning for the control and ultimate elimination of *P. vivax*. The extremes of current estimates – 15.8 million *versus* 391 million clinical cases [17,18] – emphasize the need for a validated approach to measuring the burden imposed by this important and threatening parasite.

## Additional files

Additional file 1:
**Geographic zones of relapse phenotype.** Description: Relapse patterns of strains of *P. vivax* are proposed to differ among the nine ecological zones shown above [28]. Additional file 2:
**Incidence records plotted versus the predicted MAP-based** ***Pv*** **PR values and observed concurrent** ***Pv*** **PR values. **Description: Incidence points versus MAP *Pv*PR values are shown in black and those points using concurrently measured *Pv*PR values are shown in blue. Additional file 3:
**Approximate and exact person-time shown in plots of incidence per 1,000 person-years versus parasite rate.** Description: The incidence records with concurrent *Pv*PR estimates are plotted below on linear (A) and log scales (B) below. The blue points are those with approximated person time and those in grey had exact person-time reported. Additional file 4:
**Case parasite density threshold shown in scatter plots of incidence per 1,000 person-years versus parasite rate. **Description: The incidence records are plotted below on linear (A) and log scales (B) below. The grey points are studies that used any parasitaemia in the case definition. Blue points are studies that defined a case as ≥500 parasites/μl of blood and red points, 1000 parasites/μl. Additional file 5:
**The posterior for the non-parametric fitted function giving the impact of ACD frequency on the rate of detected clinical incidence cases.** Description: A non-parametric statistical distribution the frequency of ACD was fit under a monotonicity restriction, which forces the posterior to preserve a strict ordering of the observed incidence scaling with respect to ACD frequency.
