# Supplementary figures and images for: Defining the relationship between Plasmodium vivax parasite rate and clinical disease
Source: Malar J. 2015 May 7;14:191. doi: 10.1186/s12936-015-0706-3 (PMC4429942; doi:10.1186/s12936-015-0706-3)

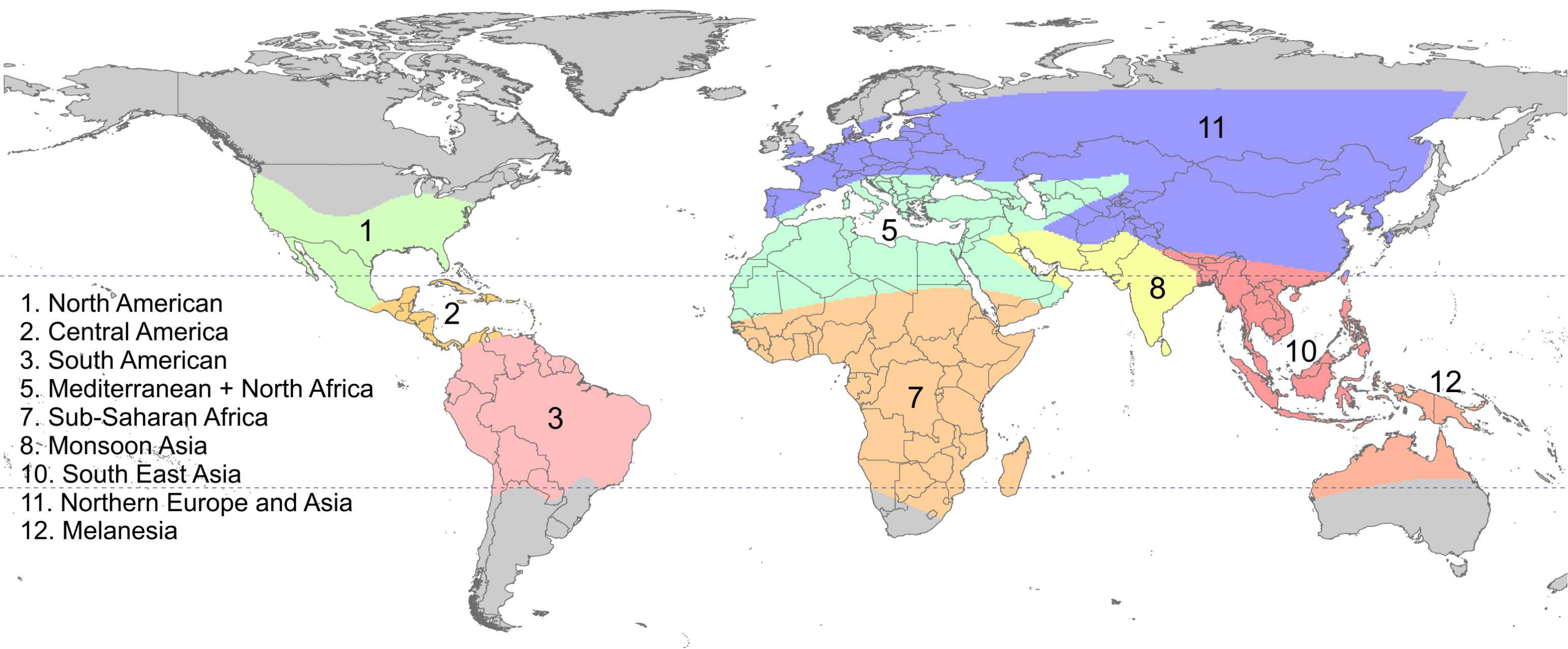

Supplement: Additional file 1: — Geographic zones of relapse phenotype. Description: Relapse patterns of strains of P. vivax are proposed to differ among the nine ecological zones shown above [28]. [file 12936_2015_706_MOESM1_ESM.png]

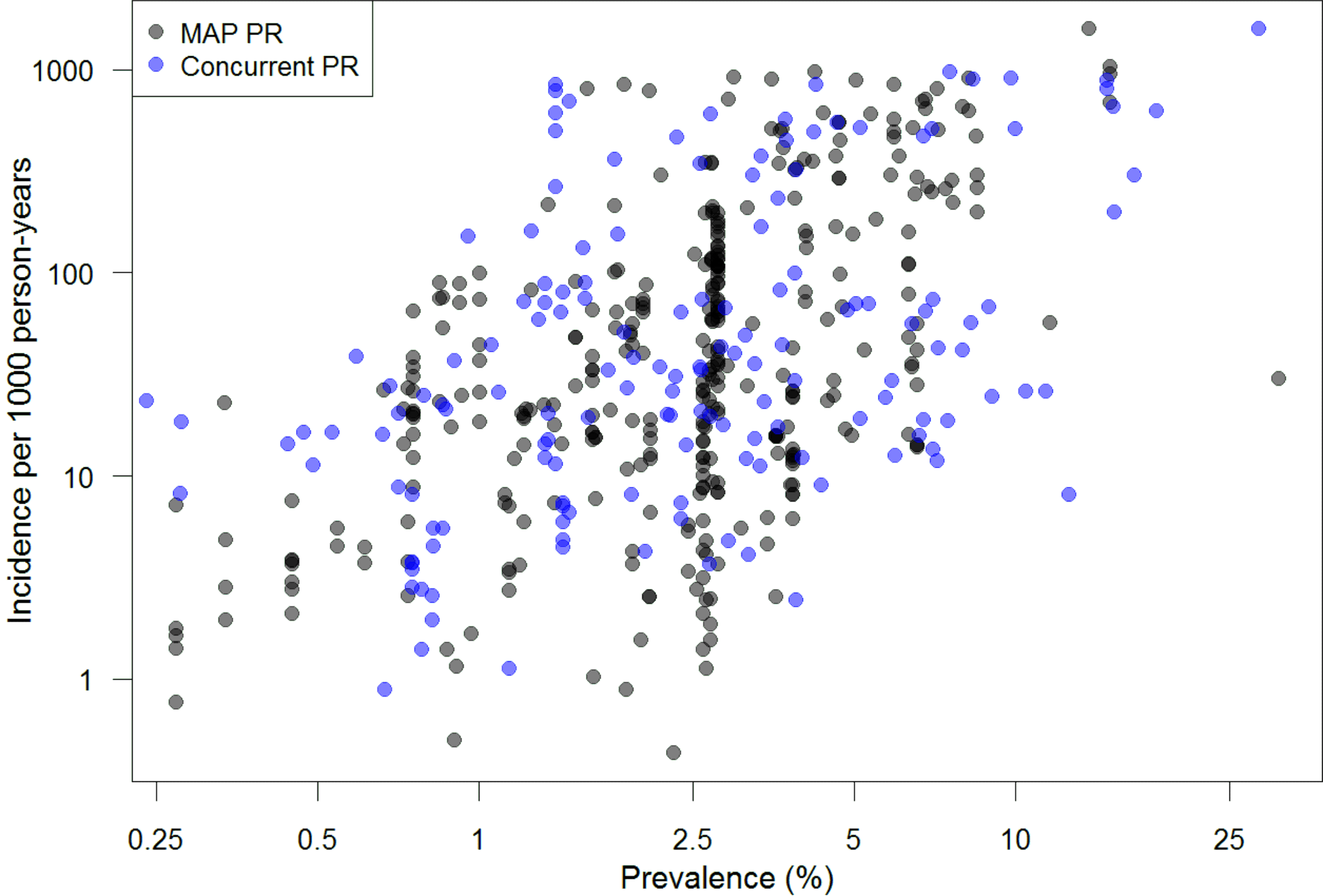

Supplement: Additional file 2: — Incidence records plotted versus the predicted MAP-based Pv PR values and observed concurrent Pv PR values. Description: Incidence points versus MAP PvPR values are shown in black and those points using concurrently measured PvPR values are shown in blue. [file 12936_2015_706_MOESM2_ESM.png]

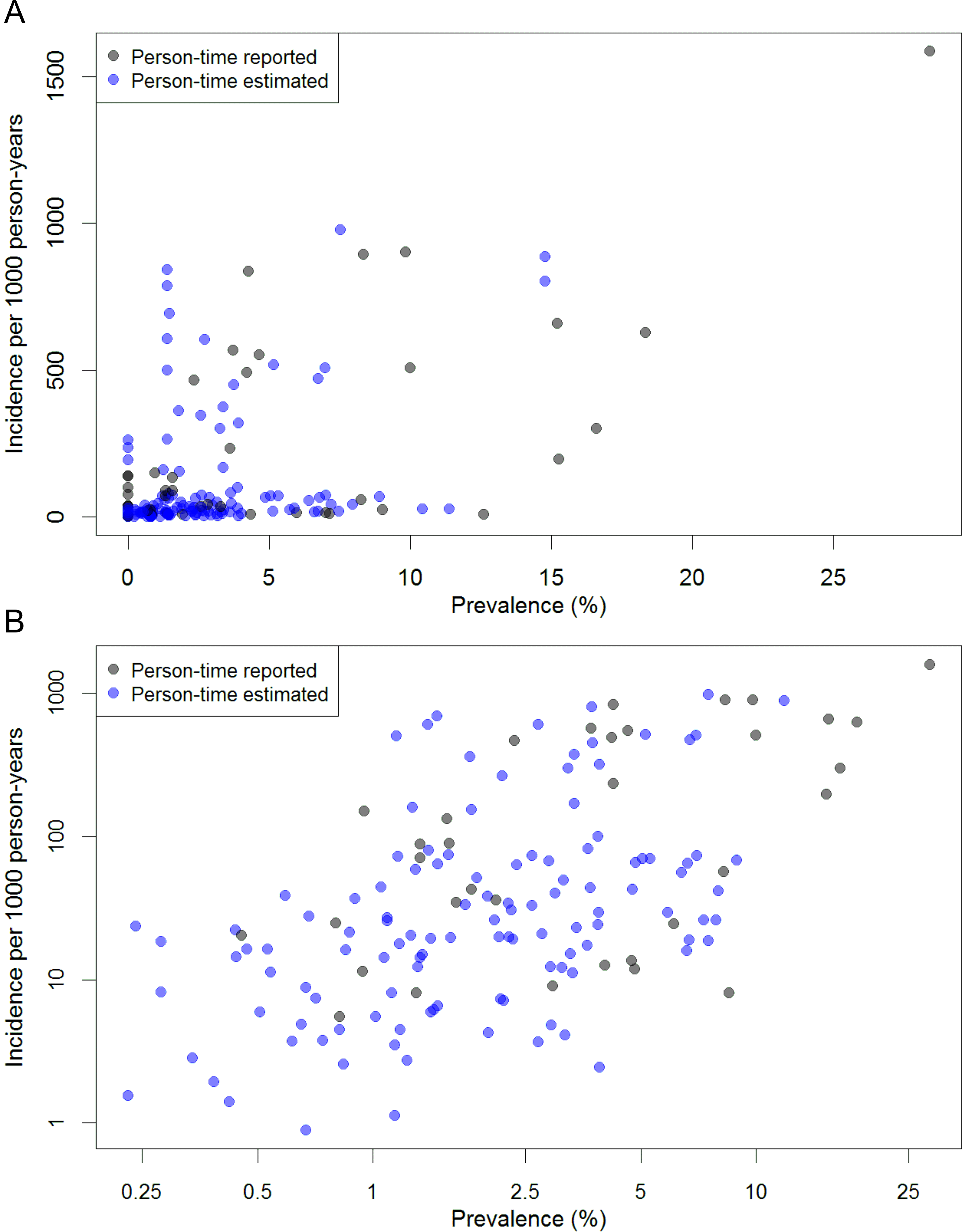

Supplement: Additional file 3: — Approximate and exact person-time shown in plots of incidence per 1,000 person-years versus parasite rate. Description: The incidence records with concurrent PvPR estimates are plotted below on linear (A) and log scales (B) below. The blue points are those with approximated person time and those in grey had exact person-time reported. [file 12936_2015_706_MOESM3_ESM.png]

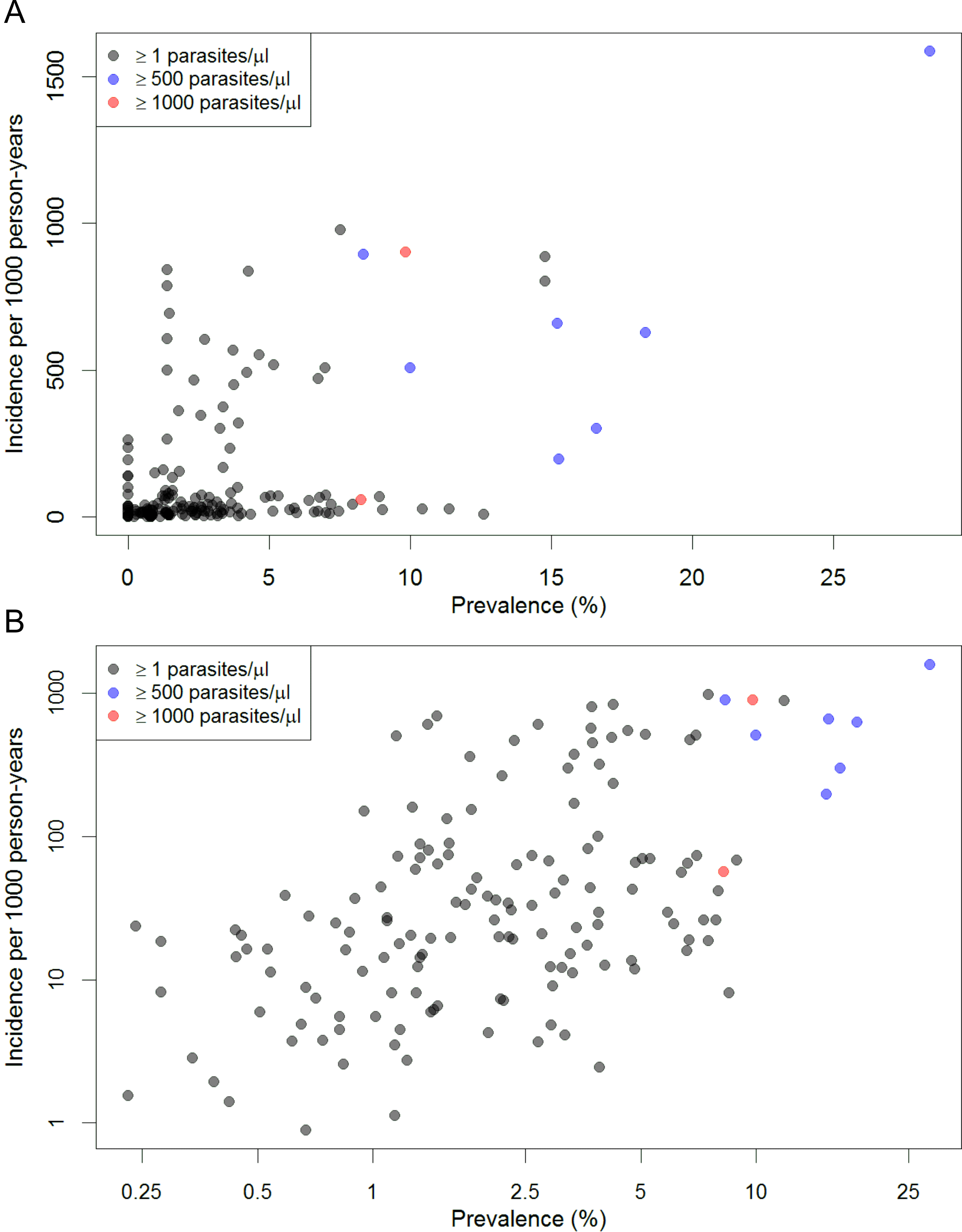

Supplement: Additional file 4: — Case parasite density threshold shown in scatter plots of incidence per 1,000 person-years versus parasite rate. Description: The incidence records are plotted below on linear (A) and log scales (B) below. The grey points are studies that used any parasitaemia in the case definition. Blue points are studies that defined a case as ≥500 parasites/μl of blood and red points, 1000 parasites/μl. [file 12936_2015_706_MOESM4_ESM.png]

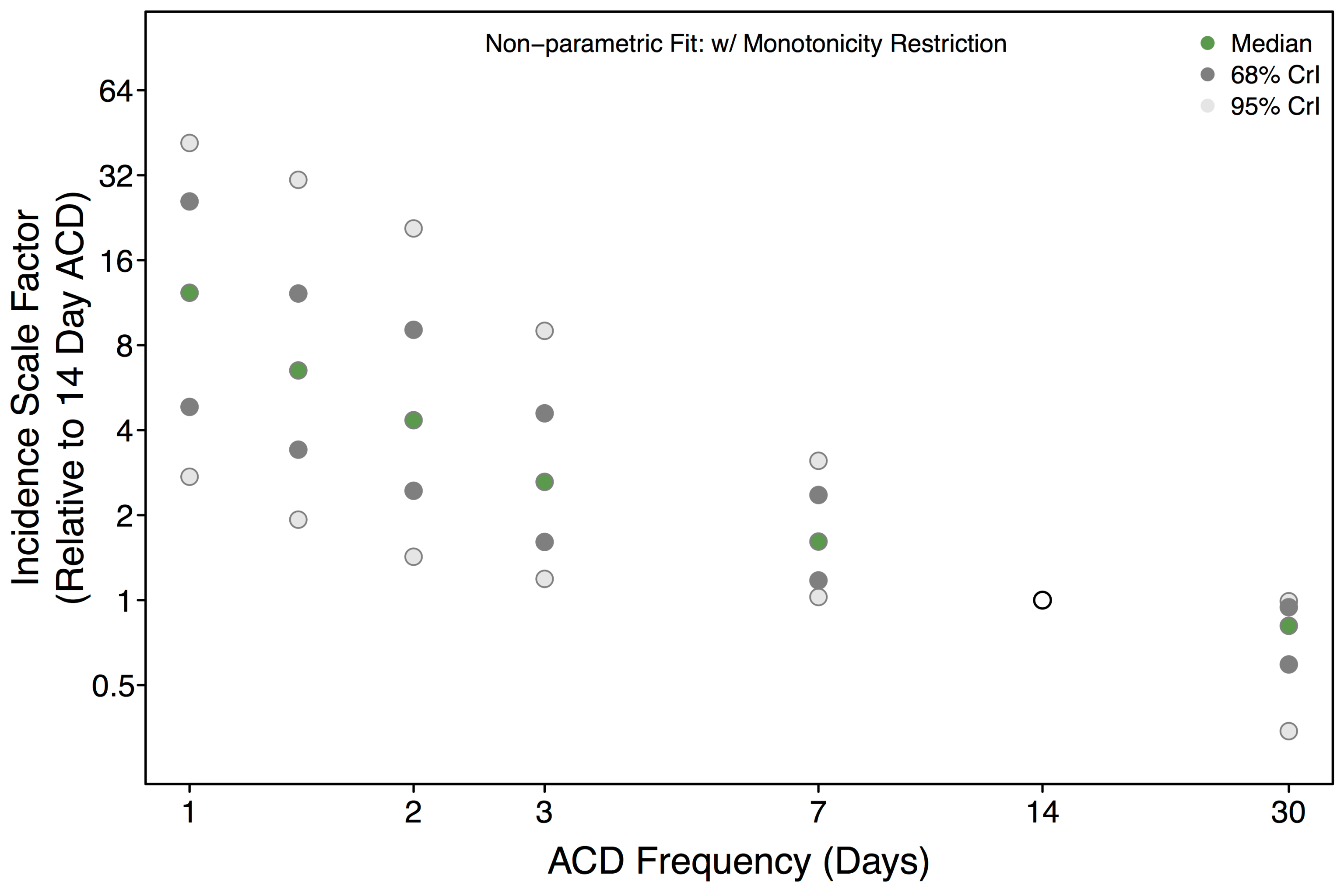

Supplement: Additional file 5: — The posterior for the non-parametric fitted function giving the impact of ACD frequency on the rate of detected clinical incidence cases. Description: A non-parametric statistical distribution the frequency of ACD was fit under a monotonicity restriction, which forces the posterior to preserve a strict ordering of the observed incidence scaling with respect to ACD frequency. [file 12936_2015_706_MOESM5_ESM.png]
